# Morbillivirus Canis Infection Induces Activation of Three Branches of Unfolded Protein Response, MAPK and Apoptosis

**DOI:** 10.3390/v16121846

**Published:** 2024-11-28

**Authors:** Santiago Emanuel Colina, Macarena Marta Williman, Marco Antonio Tizzano, María Soledad Serena, María Gabriela Echeverría, Germán Ernesto Metz

**Affiliations:** 1 Laboratorio de Virología, Centro de Microbiología Básica y Aplicada (CEMIBA), Facultad de Ciencias Veterinarias, Universidad Nacional de La Plata, La Plata CP 1900, Buenos Aires, Argentina; scolina@fcv.unlp.edu.ar (S.E.C.); mwilliman@fcv.unlp.edu.ar (M.M.W.); mtizzano@fcv.unlp.edu.ar (M.A.T.); solserena2000@fcv.unlp.edu.ar (M.S.S.); gecheverria@fcv.unlp.edu.ar (M.G.E.); 2 Consejo Nacional de Investigaciones Cientìficas y Técnicas (CONICET), CCT-La Plata, La Plata CP 1900, Buenos Aires, Argentina; 3 Agencia Nacional de Promoción de la Investigación, el Desarrollo Tecnológico y la Innovación, Godoy Cruz 2370, Ciudad Autónoma de Buenos Aires (CABA) C1425FQD, Argentina

**Keywords:** canine distemper virus, ER stress, UPR pathways, apoptosis

## Abstract

*Morbillivirus canis*, commonly named Canine distemper virus (CDV), is a morbillivirus implicated in several signs in the *Canidae* family. In dogs (*Canis lupus familiaris*), common signs of infection include conjunctivitis, digital hyperkeratosis and neuropathologies. Even with vaccination, the canine distemper disease persists worldwide so the molecular pathways implicated in the infection processes have been an interesting and promising area in new therapeutic drugs research in recent years. It is known that in the process of virus infection, the endoplasmic reticulum (ER) loses its homeostasis, inducing stress and the subsequent unfolded protein response or UPR in which three ER-trans-membrane proteins are implicated: PERK, IRE1 and ATF6. Moreover, in prolonged ER stress, the apoptosis is induced through the CHOP, as a final step of viral infection. Cell culture and molecular techniques such as RT-qPCR and RT-PCR were used in the present study. We demonstrate the activation in vitro of the three UPR pathways after infection with an attenuated strain of CDV. Also, the implication of a MAPK pathway through the p38 protein and the apoptotic CHOP was demonstrated to contribute to the process of infection. Even more, our study suggested that CDV replication occurs in a PERK-dependent manner.

## 1. Introduction

*Morbilivirus canis*, commonly known as Canine distemper virus (CDV), is the etiological agent of the disease known as canine distemper. The virus is classified within the *Morbillivirus* genus of the *Paramyxoviridae* family, which also includes the measles virus and the Peste des Petits Ruminants Virus, among others. The viral particle comprises a non-segmented negative-sense RNA, covered by a nucleoprotein (N), along with a phosphoprotein (P) and a large polymerase protein (RdRp) (L). Collectively, these form a ribonucleoprotein complex which is enclosed in an envelope containing three main glycoproteins: the fusion (F), matrix (M), and hemagglutinin (H) proteins [1,2]. The disease affects individuals of all ages within the *Canidae* family and is characterised by fever, conjunctivitis, rash, leukocytopenia and respiratory/gastrointestinal signs [3,4]. In domestic dogs (*Canis lupus familiaris*), neurological signs and digital hyperkeratosis producing hard pads are common manifestations of this disease.

Viral infections induce molecular changes that result in cell death through a variety of mechanisms, including apoptosis, necroptosis, and pyroptosis. The synthesis of substantial quantities of proteins by viruses disrupts the equilibrium of the endoplasmic reticulum (ER), thereby inducing stress and triggering a response known as the unfolded protein response (UPR) to attempt to restore ER homeostasis [5]. In case this objective is not achieved, the UPR induces cell death through apoptosis in a caspase-12-dependent manner [6,7,8,9]. The UPR comprises three distinct signalling pathways, each initiated by a transmembrane sensor protein: Protein Kinase RNA-like ER Kinase (PERK), Inositol-Requiring protein-1α (IRE1α) and Activation Transcription Factor 6 (ATF6). In normal conditions, the chaperone GRP78 (also known as Binding Immuno-globulin Protein, or BIP) is bound to the three sensors, thereby preventing the initiation of the UPR. However, when ER stress occurs, BIP separates from these proteins, thereby enabling the UPR to be initiated [10].

The PERK pathway is initiated by the dimerisation and autophosphorylation of the sensor protein. The subsequent activation of PERK enables the phosphorylation of eukaryotic translation initiation factor 2 alpha (eIF2α), which in turn induces the transcription of the Activation Transcription Factor 4 (ATF4) gene, thereby stimulating the expression of various genes, including the transcriptional factor C/EBP homologous protein (CHOP). This process is involved in the induction of cell arrest and apoptosis [11]. Furthermore, the PERK pathway is involved in the initial stages of the UPR process, where it causes a reduction in global translation [5].

The IRE1α pathway initiates in a manner that is analogous to that of the PERK. The sensor exhibits both protein kinase and endoribonuclease activities. The active form of IRE1α is capable of bonding and splicing different mRNAs, including the X-box binding protein 1 (XBP1) mRNA, also known as XBP1 unspliced (XBP1u). Consequently, this specific target is converted into an XBP1 spliced form (XBP1s), which enables the translation of an active XBP1 protein that activates genes involved in ER-assisted degradation (ERAD) to restore ER homeostasis [10,11].

The final UPR pathway involves the ATF6 sensor, which undergoes translocation to the Golgi apparatus in response to ER stress. This results in the cleavage of the sensor, yielding two distinct domains: a cytosolic N-terminal domain and a transmembrane C-terminal domain. The p50 cytosolic N-terminal domain then relocates to the nucleus, thereby inducing the activation of pro-survival genes [11]. Also, ATF6 participates in the fold capacity of ER, stimulating several GRP-proteins such as GRP34, calnexin and calreticulin [10].

CDV has been demonstrated to induce apoptosis in a range of cell lines, including the green monkey epithelial cell line (VERO) and the human colon cancer cell line (HCT-15) [12,13,14]. The role of ER stress in CDV pathogenesis has been the subject of considerable debate. Following infection with a recombinant CDV, Brunner and colleagues demonstrated that both the calreticulin chaperone and the CHOP transcription factor are upregulated, suggesting a potential involvement of the unfolded protein response pathway [15]. It has been demonstrated that differences in CDV strains induce the activation of the same signalling pathways in ER stress conditions, although with varying degrees of intensity [16].

The present study examines the role of ER stress, with a particular focus on the UPR pathways, and its implications in the context of in vitro CDV infection processes. The objective of this study is to investigate the potential induction of ER stress and the subsequent activation of the three branches of the UPR, as well as the cell death that occurs following the induction of apoptosis.

## 2. Materials and Methods

### 2.1. Cell Culture, Virus and Reagents

The African green monkey (VERO) CCL-81 cell line, employed in all experiments, was obtained from Asociación Banco Argentino de Células (ABAC). The cells were cultured in minimum essential medium (MEM) supplemented with 10% (*v*/*v*) irradiated foetal bovine serum (FBS), gentamicin 0.04 mg/mL and amphotericin 1.25 mg/mL, and incubated at 37 °C with 5% CO_2_ in order to facilitate optimal cell growth. The attenuated CDV Onderstepoort strain (CDVOnd) employed in all assays was kindly provided by Dr. Marco Antonio Tizzano. Tunicamycin (Cell Signaling Technology Inc., Danvers, MA, USA, #12819) was employed as a positive control for ER stress induction at a final concentration of 7.5 μg/μL. The PERK inhibitor, GSK2606414, was obtained from Thermo Fisher Scientific (Waltham, MA, USA, #5165355MG) and was used in a final concentration of 400 nM.

### 2.2. Viral Stock and Titration Assay

The CDVOnd strain was cultivated in VERO CCL-81 cells. The cells were incubated for one hour at 37 °C to facilitate viral adsorption. Next, the viral supernatant was removed, and the cells were supplemented with MEM and 2% FBS. The infected cells were maintained at 37 °C until several cytopathic effects (CPEs) were observed. Thereafter, the supernatant was collected, aliquoted and frozen at −70 °C. Viral titration and the determination of TCID_50_ was assayed using the Reed and Muench method (Supplementary Materials and Methods 1) [17].

### 2.3. Infection Experiments

Vero CCL-81 cells were cultivated until they reached confluence in a 12-well plate. The infection was performed at a multiplicity of infection (MOI) of 1 and initially all analyses were performed at multiple hours post infection (hpi) (8, 16 and 24 hpi). Briefly, the infected cells were incubated for one hour at 37 °C with gentle agitation. Later, the cells were supplemented with MEM and 2% FBS, and the plate was incubated at 37 °C in a 5% CO_2_ atmosphere. Mock cells and cells induced with tunicamycin were prepared in a similar manner, at the maximum time point assayed, to serve as negative and positive controls, respectively.

### 2.4. RNA Extraction and Reverse Transcription Assay

Following each time point, the cells were harvested and the total cellular RNA was extracted using the High Pure RNA Isolation Kit from Roche (Indianapolis, IN, USA), in accordance with the manufacturer’s instructions. After that, 1 μg of total RNA was retro-transcribed using a random hexamer from Biodynamic SRL (Buenos Aires, Argentina) and M-MLV Reverse Transcriptase from Promega Corporation (Madison, WI, USA). The cDNA obtained was stored at −20 °C for further analysis.

### 2.5. PCR Assay

Polymerase chain reactions (PCRs) were conducted for each condition using primers that enabled the differentiation of XBP1 mRNA splicing, as well as the detection of unspliced transcripts. This process involved the removal of a 26-nucleotide intron, thereby facilitating the translation of a functional protein [10]. The sequence for each primer was as follows: XBP1-Fw: 5′-CCTTGTAGTTGAGAACCAGG-3′ and XBP1-Rv: 5′-GGGGCTTGGTATATATGTGG-3′ [18]. The PCR mix reaction was conducted in a final volume of 20 μL using Inhibitor-resistant DNA polymerase Kit T-Plus 500 U and the dNTPs in 100 mM from Inbio Highway SRL. The concentration of Taq Polymerase was 0.125 U/μL, the dNTPs’ concentration was 250 nM and each primer was 250 nM. A volume of 1 μL of cDNA was used as a template. PCR conditions comprised an initial denaturation step at 95 °C for 5 min, 35 cycles of 94 °C, 55 °C and 72 °C for 30 s each, and a final extension step at 72 °C for 5 min. Band densitometry was performed using the Gel-Pro Analyzer 4.0 software, with GAPDH used for the normalisation of the bands. The PCR products were separated on a 2% agarose gel and visualised by ultraviolet light after staining with ethidium bromide.

### 2.6. qPCR Assay

Quantitative polymerase chain reactions (qPCRs) were conducted with the use of iQ SYBR Green Supermix from Bio-Rad (Hércules, CA, USA), in accordance with the instructions provided by the manufacturer. The final reaction volume was 10 μL. The primer pairs are presented in Table 1, and GAPDH was employed as a housekeeping gene to normalise the transcript levels using the 2^−ΔΔCT^ method. To validate the amplification results, the melting curves were analysed and the products were resolved by 1.5% agarose gel electrophoresis, as described above.

### 2.7. Inhibition of PERK Pathway

To corroborate the importance of the PERK pathway for the viral progeny of CDVOnd, a classical selective inhibitor, GSK2606414 (GSK), was employed at a concentration of 400 nM. The VERO CCL-81 cells were incubated for one hour in the presence of GSK. Following the removal of the inhibitor, the cells were infected at MOI 1 for 1 h. Finally, the viral inoculum was replaced with a maintenance medium in the presence of the inhibitor and the supernatant was harvested at different time points (48, 72 and 96 hpi). To enable a comparison of the viral progeny with and without GSK, a TCID_50_ assay using the Reed and Muench method was performed as explained before. Additionally, viral loads were assayed by qPCR assay to correlate with viral titration.

### 2.8. DNA Fragmentation Assay

The induction of apoptosis can be evaluated through a specific process that involves the production of DNA fragments of a defined size. In the final stages of apoptosis, DNA is processed by an endonuclease, resulting in the generation of multiple fragments. These can be visualised in agarose gel as a ladder-like pattern [24]. The production of DNA fragments was therefore evaluated following 16, 24, 48 and 72 hpi with the CDVOnd strain. Mock cells were analysed at the final time point of 72 h. The VERO CCL-81 cells were prepared in a 24-well plate. Following infection, genomic DNA was obtained by the phenol:chloroform:isoamyl alcohol extraction method (Supplementary Materials and Methods 2) and separated by 1% agarose gel electrophoresis and visualised with GelGreen 10,000× from Biotium (Fremont, CA, USA) in the Safe Imager 2.0 transilluminator from Invitrogen.

### 2.9. Statistical Analysis

The RT-PCR and qPCR experiments were conducted in triplicate and subsequently analysed using the GraphPad Prism 8 software. The results are presented as the mean ± standard deviation (SD). In all experiments, the unpaired Student’s *t*-test was used to analyse the statistical differences for comparisons between two groups. A *p* value < 0.05 was considered statistically significant. Asterisks in figures indicate statistical significance (* *p* < 0.05, ** *p* < 0.01, *** *p* < 0.001 and **** *p* < 0.0001).

## 3. Results

### 3.1. ER Stress Induced by CDV Infection

**Figure 1:**
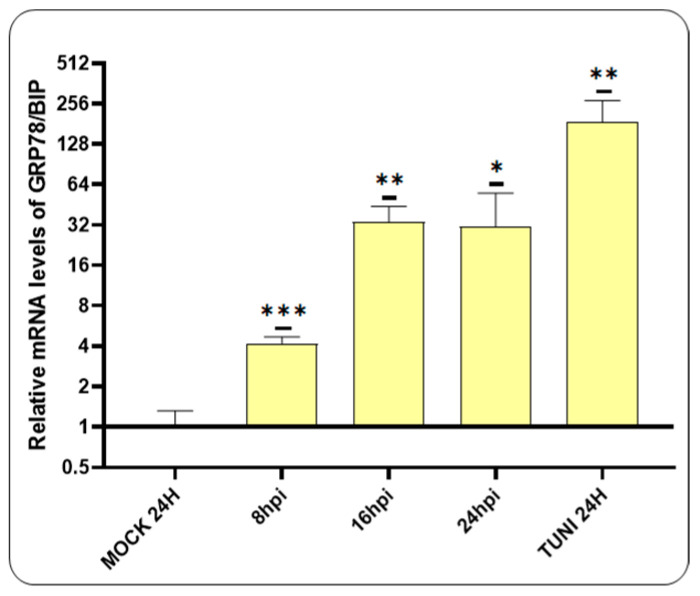
Activation of BIP in VERO CCL-81 cells. An RT-qPCR assay was conducted to evaluate the expression of the GRP78/BIP transcript following the infection with CDVOnd. The induction of ER stress was detected at 8 hpi, reaching its maximum value at 16 hpi. The levels of BIP mRNA were relativised to the mock experiment and represented as the mean ± standard deviation (SD) of three independent experiments. For statistical significance, * *p* < 0.05, ** *p* < 0.01 and *** *p* < 0.001.

The titer of the CDVOnd strain used in our experiment was 10^4.5^ TCID_50_/50 µL. An RT-qPCR assay was therefore performed to investigate the generation of ER stress following CDV infection, with an initial focus on GRP78/BIP transcript levels (Figure 1).

Our findings revealed that the mRNA levels of BIP were significantly elevated following CDV infection. The initial detection occurred at 8 hpi, with a subsequent eight-fold increase at 16 hpi, which was maintained at 24 hpi.

### 3.2. CDV Infection Activates PERK Pathway

**Figure 2:**
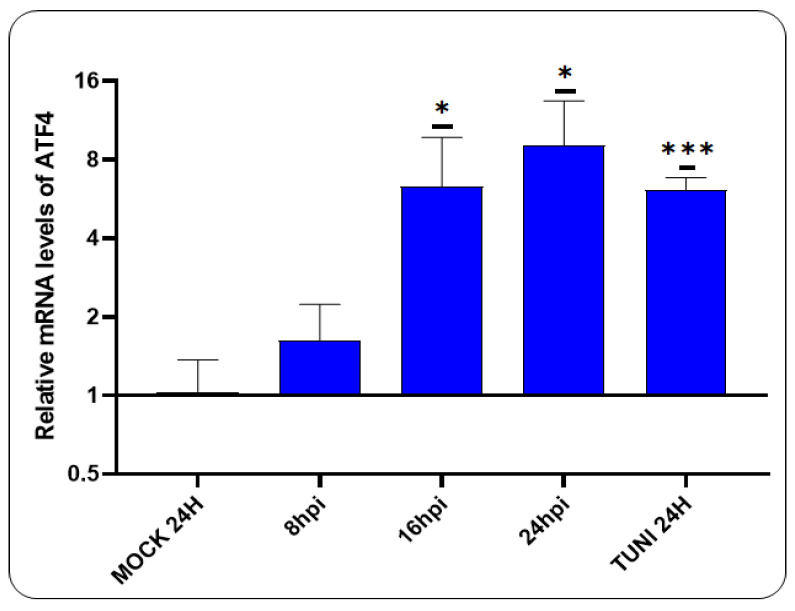
Activation of the PERK pathway in CDVOnd infection. The relative levels of the ATF4 transcript indicate the activation of the PERK pathway following 16 hpi with the CDVOnd strain. The data were relativised to the mock well and are presented as means ± SD from three independent experiments. Statistical significance * *p* < 0.05, *** *p* < 0.001.

Having previously demonstrated that CDVOnd infection induces ER stress, we now turn our attention to the PERK pathway (Figure 2).

A notable elevation in ATF4 mRNA levels was detected following 16 hpi, exhibiting a magnitude comparable to that observed in tunicamycin-induced ER stress cells.

### 3.3. Activation of ATF6 Pathway After CDV Infection

**Figure 3:**
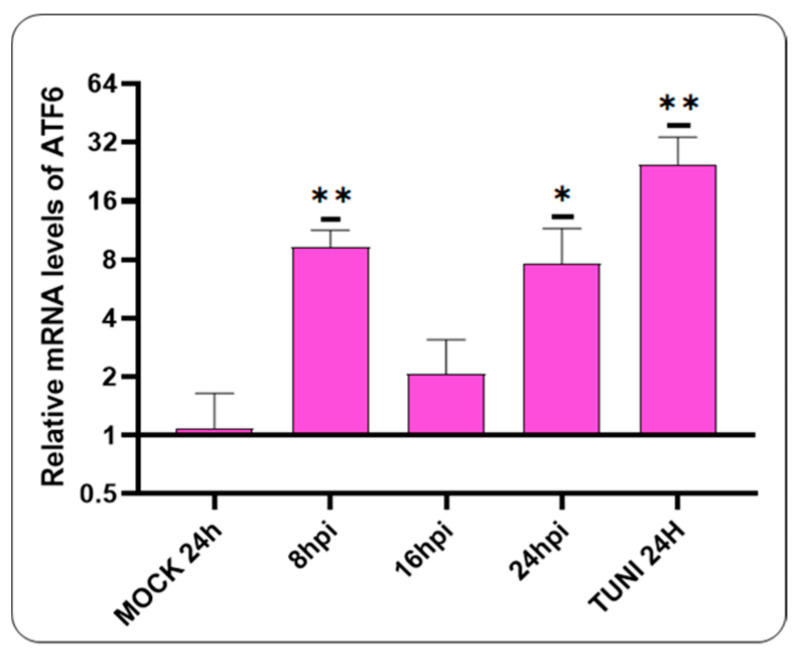
The ATF6 pathway is activated by the CDVOnd strain. The ATF6 mRNA levels were detected as early as 8 hpi. All values were relativised to the mock experiment. The values are presented as means ± SD of three independent experiments. Statistical significance values are indicated by * *p* ˂ 0.05 and ** *p* ˂ 0.01.

The subsequent objective is to investigate the potential for ATF6 pathway activation following a CDVOnd infection (Figure 3).

Our data showed the activation of the ATF6 pathway at the earliest stages of CDV infection, specifically at 8 hpi. At 16 hpi, a decline in mRNA levels was observed. However, at 24 hpi, an elevation in mRNA levels was evident.

### 3.4. IRE1⍺ Pathway Is Activated by CDVOnd Infection

**Figure 4:**
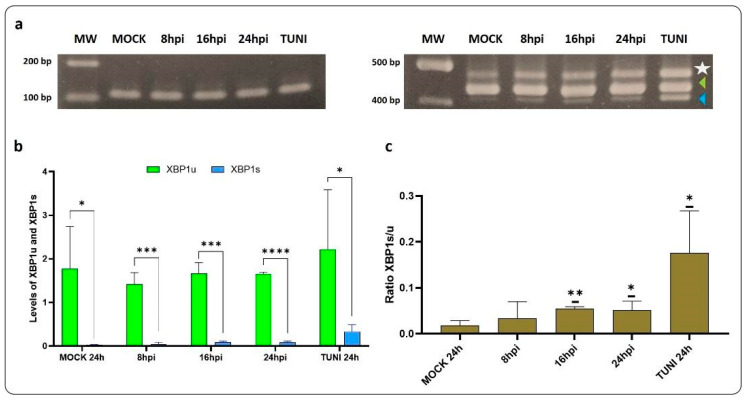
XBP1 spliced after CDVOnd infection. (**a**) The images displayed are representative photographs of 2% agarose gels with GAPDH (**left**) and XBP1 (**right**) bands for each condition from the same experiment. The triangles indicate the XBP1 transcripts, XBP1u (green) and XBP1s (blue) with a size of approximately 440 bp and 414 bp, respectively. The star symbol denotes a hybrid between the two forms of XBP1 (XBP1H), with an apparent size of 466 base pairs. (**b**,**c**) Band densitometry assay. The expression levels of XBP1 species were relativised using a value of 1 for the GAPDH bands. In (**b**), a comparison was made between the levels of XBP1u and XBP1s. In (**c**), the ratio XBP1s/XBP1u relativised to the mock condition was evaluated in each condition. Levels are represented as means ± SD of three independent experiments. In all cases, statistical significance is indicated by * *p* < 0.05, ** *p* < 0.01, *** *p* < 0.001 and **** *p* < 0.0001.

The splicing of the XBP1 transcript was evaluated through the use of RT-PCR and band densitometry assay (Figure 4).

The results of this experiment demonstrated that the ratio of XBP1s/XBP1u was increased after 16 hpi in comparison with the mock control. In addition, a third band was visualised in our study, which corresponds to a hybrid between the two XBP1 forms [21,25]. Therefore, we confirm that the IRE1α pathway is activated in vitro following CDV infection.

### 3.5. CDVOnd Infection Induces Apoptosis in VERO CCL-81

**Figure 5:**
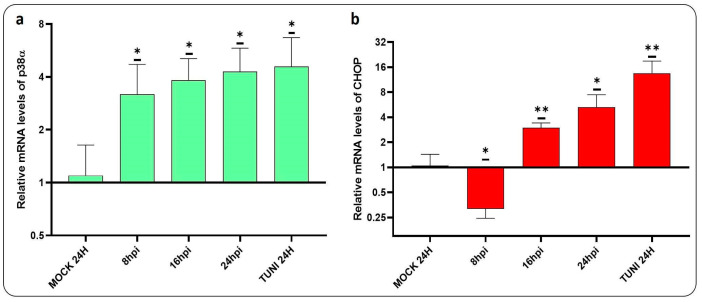
p38⍺ and CHOP induced by CDVOnd infection. The figure illustrates the relative levels of mRNA transcripts for two transcription factors involved in the activation of apoptotic genes. (**a**) Levels of the p38α mRNA transcript were found to be upregulated following CDV infection. (**b**) Levels of the CHOP transcript in response to CDV infection, illustrating the upregulation of this gene. The data were relativised to the mock experiment and expressed as means ± SD from three independent experiments. Statistical significance was determined using the following criteria: * *p* < 0.05, ** *p* < 0.01.

In order to gain insight into the mechanisms underlying apoptosis induced by CDV infection, we have conducted a study to assess the levels of p38α and the CHOP mRNA transcript (Figure 5).

**Figure 6:**
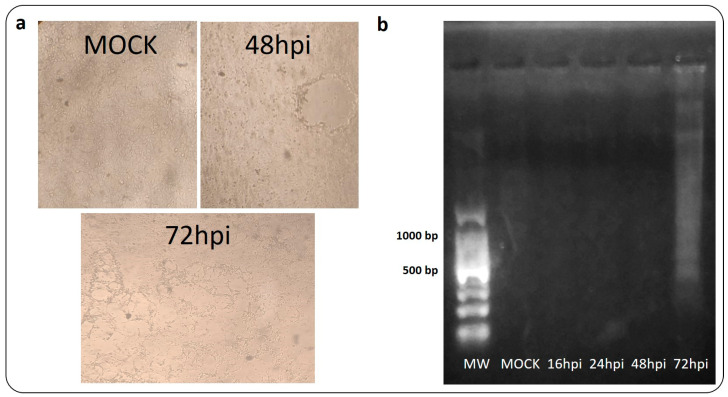
DNA laddering experiment after CDV infection. The study of apoptosis was conducted through the generation of DNA ladder-like patterns over a period of 16, 24, 48 and 72 h following the infection of CDVOnd. A 100 bp ladder (MW) was employed as a weight molecular marker. (**a**) Photographs taken at 4X of the mock and 48 hpi (**up**) and 72 hpi (**down**) wells. (**b**) Electrophoretic analysis of genomic DNA in a 1% agarose gel stained with GelGreen.

Both transcripts involved in apoptosis induction were found to be upregulated. Activation of p38α could be observed at 8 hpi, while activation of the CHOP occurred at 16 hpi. Additionally, the progression of apoptosis was investigated via the DNA fragmentation assay (Figure 6).

The infected-VERO CCL-81 monolayers exhibited an evident CPE at 72 hpi. Extraction of DNA from these infected cells demonstrated the characteristic DNA laddering at 72 hpi.

### 3.6. Viral Replication After CDVOnd Infection

**Figure 7:**
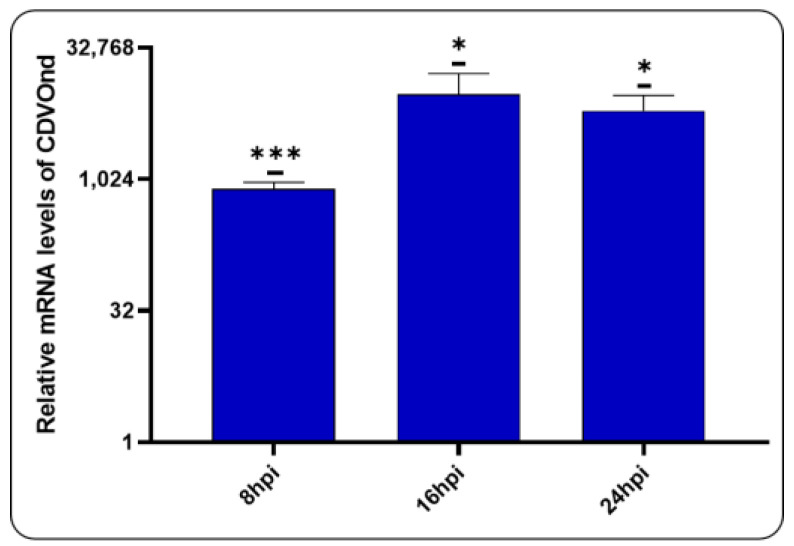
Production of viral RNA following CDV infection. The relative RNA level of the CDVOnd strain was investigated using primers to the N gene at different time points. The levels of the viral genome were relativised to the mock experiment and data are presented as means ± SD of three independent experiments. For a statistical significance: * *p* ˂ 0.05, *** *p* ˂ 0.001.

In order to evaluate the production of viral RNA following infection, RT-qPCR assay was performed using specific N gene primers (Figure 7).

The results demonstrated the production of viral RNA following CDV infection. Viral RNA levels increased at 8 hpi, a process that continued at 16 and 24 hpi.

### 3.7. PERK Inhibition and Viral Quantification

**Figure 8:**
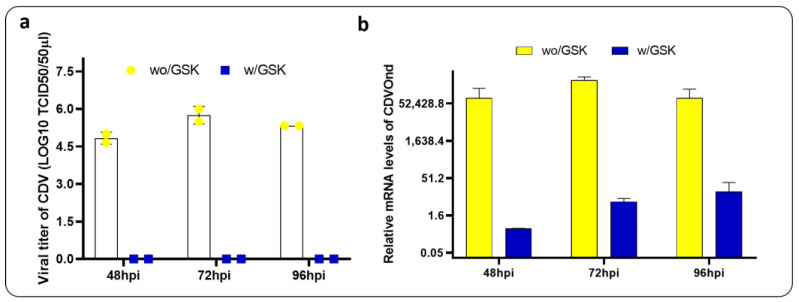
Inhibition of the PERK pathway affects viral progeny. Both experiments were conducted in duplicate in two conditions: without the PERK inhibitor (wo/GSK) and with the PERK inhibitor (w/GSK). (**a**) The TCID_50_ assay was performed to quantify CDVOnd progeny at 48, 72 and 96 hpi. (**b**) Viral load performed by qPCR targeted the N gene of CDV. The mRNA levels were relativised to mock conditions.

The subsequent objective was to evaluate the potential impact of inhibiting the UPR pathway on the viral progeny at different time points. To this end, we have chosen to inhibit the PERK pathway with the chemical inhibitor GSK2606414 and to compare the viral titer and load in two conditions, with and without an inhibitor, at several time points (Figure 8).

The viral titer obtained in the presence of the PERK inhibitor was markedly diminished, with no virions discernible by titration. Conversely, means of 10^5.5^ TCID50 were obtained in the absence of PERK pathway inhibition. Similar observations were obtained by qPCR viral load quantification.

## 4. Discussion

It is well documented that the unfolded protein response pathways are involved in the pathogenesis of various diseases, including cancer, diabetes and viral infections [26,27]. Viruses play a significant role in the modulation of cell survival. In the case of RNA viruses, it has been demonstrated that they interact with the endoplasmic reticulum during their replication processes [5]. The ER chaperone protein GRP78/BIP plays a pivotal role in the activation of ER stress sensors and has recently been identified as a crucial protein in the context of viral infection [28,29].

Our investigation revealed that BIP expression is upregulated following CDVOnd infection (Figure 1). Previous research had also identified an increase in BIP expression at varying levels when utilising a wild-type and attenuated CDV strain [16]. This activation has been observed in other paramyxovirus infections such as Newcastle Disease Virus (NDV) and Respiratory Syncytial Virus (RSV) and in this last case, in a dose-dependent manner [30,31]. Furthermore, the expression of the H protein from two distinct morbillivirus strains was sufficient to elevate BIP levels at early stages of infection [16,32]. This emphasises the significance of this chaperone in morbillivirus infection and in a wide range of other viral families [33].

Following the induction of ER stress, the UPR mechanism initiates a restoration of lost homeostasis through the action of three transmembrane proteins. A primary response involves the activation of the PERK pathway, which results in the activation of the eIF2α, promoting pro-adaptive signalling mechanisms that inhibit protein synthesis and activate the expression of ATF4 [10]. In this study, we found the activation of the PERK pathway, as indicated by the increase in ATF4 mRNA levels (Figure 2). At 16 hpi, we observed a statistically significant upregulation in ATF4 mRNA in cells infected with CDVOnd, comparable to the levels observed in cells treated with tunicamycin. Similar results were obtained with RSV, as evidenced by the activation of eIF2α [30]. Furthermore, our findings revealed the activation of the ATF6 branch of the UPR as early as 8 hpi (Figure 3), which is consistent with the observations made in prior studies where this pathway was also activated [30]. Moreover, the implication of the ATF6 pathway was demonstrated in the induction of autophagy following infection with the Peste des Petits Ruminants Virus, a member of the Morbillivirus genus [34].

In accordance with the proposed ER stress pathway, the activation of the IRE1 branch results in the cleavage of XBP1 mRNA (XBP1u) into its spliced form (XBP1s) [6,10]. Here, the ratio of XBP1s/XBP1u indicated that the maximum rate of XBP1s was reached at 16 hpi (Figure 4). Nevertheless, evidence suggests that ATF6 plays a role in XBP1 modulation, activating its transcription [35,36]. Therefore, the activation of ATF6 at 8 hpi may contribute to the transcription of XBP1, as evidenced by the higher level of XBP1u observed at later time points. It would be interesting to analyse the regulation of the IRE1-dependent decay of mRNA (RIDD) after CDVOnd infection to examine the sole activation of the IRE1 pathway.

The different branches of the UPR activated by ER stress have the capacity to regulate the equilibrium between viral infection and cellular fate. The death of cells following prolonged ER stress has been documented in the context of infection by several viruses [5]. The transcription factor C/EBP homologous protein (CHOP), also known as growth arrest and DNA damage-inducible gene 153 (GADD153), has been demonstrated to mediate apoptosis in cells under ER stress [37]. Furthermore, the IRE1 pathway has been implicated in MAPK activation and this pathway has been shown to participate in CHOP expression during tunicamycin-induced apoptosis, indicating that this interaction is crucial for innate immunity in mammals [38,39,40]. The results presented here demonstrate that CDVOnd infection clearly activates p38α kinase, with the amount of mRNA detected after 24 hpi being equivalent to that obtained with tunicamycin (Figure 5a). Furthermore, the induction of apoptosis following ER stress induced by the infection was evidenced by the upregulation of the CHOP at 16 hpi (Figure 5b). It is noteworthy that our findings evidenced a reduction in CHOP expression at 8 hpi. The PERK-eIF2α-ATF4 pathway is the primary promoter of the CHOP, with its function capable of switching from being anti-apoptotic to pro-apoptotic depending on the duration of the ER stress [41]. Therefore, it can be hypothesised that the poor upregulation of ATF4 at 8 hpi may have been involved in the downregulation of the CHOP transcript at early times of infection.

Viral load analysis conducted at times below 24 hpi revealed the transcription of the CDV N gene (Figure 7). However, the presence of virions could not be determined by titration. Quantification of virions in the cell supernatant was only possible at times exceeding 48 hpi (Figure 8a).

It is therefore proposed that CDVOnd initiates its replicative cycle by transcribing basal levels of the N gene at 8 hpi. These levels increase significantly at 16 hpi (Figure 7) and are less evident at later infection times (Figure 8b). This would suggest that the onset of the ER stress may be related to the high levels of N mRNA detected. However, it remains to be analysed whether other genes are involved, as demonstrated with the H gene [16].

It has been demonstrated that viral infections regulate ER stress by influencing the equilibrium between cell survival and death by apoptosis [42]. The preliminary findings of this study propose that CDVOnd may exploit the inhibition of the apoptosis process at early stages to transcribe its genome. However, at late stages of infection, DNA fragmentation was identified as an indicator of the apoptosis process (Figure 6b). Additionally, other researchers have observed similar outcomes with the attenuated Onderstepoort strain [12].

It is notable that the inhibition of the PERK pathway resulted in a substantial reduction in N mRNA levels, with values that were even lower than those observed at 8 hpi in the absence of PERK inhibition (Figure 8b). Moreover, infectious virions could not be detected by titration in the presence of a PERK pathway inhibitor (Figure 8a).

We concluded that, in the event of the inhibition of the PERK pathway, the attenuating control of protein translation provided by this pathway will no longer be available. Consequently, the IRE1 and ATF6 pathways will be balanced towards protein degradation, thus preventing the assembly and formation of new CDV virions, as has been observed in the cases of other viruses [43,44].

## 5. Conclusions

The findings of this study demonstrate that the Onderstepoort strain of *Morbillivirus canis* activates the three sensors of UPR following the induction of ER stress. Furthermore, the MAPK pathway, through the p38α protein and the CHOP transcription factor, which is involved in the apoptosis process, was demonstrated to be implicated in the infection events. The present study focused on the assessment of mRNA levels, while the evaluation of protein expression was identified as a topic for further investigation. The inhibition of the PERK pathway provided compelling evidence that this UPR branch plays a significant role in viral progeny at later stages of infection, indicating a PERK-dependent replication mechanism.

The design of new molecular compounds against these pathways could be studied in an attempt to identify novel antiviral agents for the treatment of canine distemper disease, a pathology that continues to affect domestic and non-domestic animals globally.

## Figures and Tables

**Table 1:** List of primers used in RT-qPCR experiments.

Use	Gene	Sequence (5′ to 3′)	Size (bp)	Reference
House-	GAPDH-Fw	AGGTCGGAGTCAACGGATTT	112	[18]
keeping	GAPDH-Rv	TAGTTGAGGTCAATGAAGGG	112	[18]
ER stress	BIP-Fw	ACCGCTGAGGCTTATTGGG	147	[18]
activation	BIP-Rv	TGCCGTAGGCTCGTTGATG	147	[18]
PERK	ATF4-Fw	CCAACAACAGCAAGGAGGAT	143	[19]
pathway	ATF4-Rv	GTGTCATCCAACGTGGTCAG	143	[19]
ATF6	ATF6-Fw	CGAATAGCCCAGTGAA	180	[20]
pathway	ATF6-Rv	ATCTCGCCTCTAACCC	180	[20]
Apoptosis	CHOP-Fw	ACCAAGGGAGAACCAGGAAACG	201	[21]
Apoptosis	CHOP-Rv	TCACCATTCGGTCAATCAGAGC	201	[21]
MAPK	p38α-Fw	GCCCAAGCCCTTGCACAT	156	[22]
pathway	p38α-Rv	TGGTGGCACAAAGCTGATGAC	156	[22]
Viral	CDV-N-Fw	TTCTGAGGCAGATGAGTTCTTC	829	[23]
genome	CDV-N-Rv	CTTGGATGCTATTTCTGACACT	829	[23]

## Data Availability

All data can be requested from the corresponding authors.

## Supplementary Materials

The following supporting information can be downloaded at: https://www.mdpi.com/article/10.3390/v16121846/s1: Supplementary Material and Method 1. TCID50 assay using Reed and Muench method; Table S1: Example of the table used to register positive/negative wells in each dilution; Table S2: Calculation of TCID50 by the Reed-Muench method. Supplementary Material and Method 2. Phenol:Chloroform:Isoamyl alcohol extraction method.
