# Supplementary material for: Morbillivirus Canis Infection Induces Activation of Three Branches of Unfolded Protein Response, MAPK and Apoptosis
Source: Viruses. 2024 Nov 28;16(12):1846. doi: 10.3390/v16121846 (PMC11680218; doi:10.3390/v16121846)
Supplement: Supplementary file 1 [file viruses-16-01846-s001.zip › viruses-3286015-supplementary.pdf]

Supplementary Material and Method 1. $TCID_{50}$ assay using Reed & Muench method.

Reed & Muench endpoint dilution method is a quantitative technique that determines the amount of virus required to produce a cytopathic effect in 50% of inoculated tissue culture cells.

The viral supernatants derived from each experimental condition were fractionated and stored at -70°C. To determine the titer, each sample was subjected to serial dilution in MEM medium, with the dilution factor ranging from 10^{-1} to 10^{-7}. Four replicates of 50 µl of each dilution were seeded in 96-well plates and subsequently added 50.000 Vero CCL-81 cells per well. Replicates replacing viral dilution with 50 µl of MEM medium were used as cell viability control (CVC).

Then, the plate was incubated at 37°C in a CO_2 incubator and observed daily under a microscope. In each dilution, the wells exhibiting cytopathic effects (CPE) were recorded in a table until the same results were registered for consecutive days (Table S1).

Table S1: Example of the table used to register positive/negative wells in each dilution.

	**Viral titration**			
	**well 1**	**well 2**	**well 3**	**well 4**
**CVC**	OK	OK	OK	OK
**dil -7**	-	-	-	-
**dil -6**	-	-	+	-
**dil -5**	+	-	+	+
**dil -4**	+	+	+	+
**dil -3**	+	+	+	+
**dil -2**	+	+	+	+
**dil -1**	+	+	+	+

In order to calculate the dilution containing 50% of the wells with CPE, a table was made according to Reed & Muench (Table S2). The proportionate distance (PD) between the two dilutions that are closest to 50% positive was calculated using the following formula: The proportionate distance (PD) is calculated as follows:

Table S2: Calculation of $TCID_{50}$ by the Reed-Muench method.

	**Dil -1**	**Dil -2**	**Dil -3**	**Dil-4**	**Dil-5**	**Dil-6**	**Dil-7**
**Observed positive wells**	4/4	4/4	4/4	4/4	3/4	1/4	0/4
**Observed negative wells**	0/4	0/4	0/4	0/4	1/4	3/4	4/4
**Cumulative positives ←**	20	16	12	8	4	1	0
**Cumulative negatives→**	0	0	0	0	1	4	8
**Positives/positives +negatives**	20/20	16/16	12/12	8/8	4/5	1/5	0/8
**% positive in each dilution**	100%	100%	100%	100%	80%	20%	0%

PD = (positive above 50% - 50%) / (positive above 50% - % positive below 50%)

The proportionate distance (PD) is then applied to the dilution that produced a percentage of infection immediately below 50 percent. The reciprocal of this dilution represents the number of $TCID_{50}$ in 50 µl of the viral inoculum, which is then converted to $TCID_{50}$/ml

Supplementary Material and Method 2. Phenol:Chloroform:Isoamyl alcohol extraction method.

VERO CCL-81 cells were seeded in a 24-well plate and incubated ON at 37°C in 5% CO2. After 1 h of viral infection at MOI 1, cells were incubated until harvested at the different time points tested: 16, 24, 48, 72 hpi, and the MOCK well was assayed at the last time point of the experiment. The cells were resuspended in 500 µl BPS and mixed with one volume of phenol:chloroform:isoamyl alcohol (25:24:1). The mixture was gently vortexed and centrifuged at 12000 rpm for 15 min. The aqueous phase was transferred to a microcentrifuge tube and a second round of extraction was carried out with chloroform:isoamyl alcohol (24:1).

The aqueous phase was then mixed with 18 µl of sodium acetate (3 M) and 2 volumes of absolute ethanol. The genome was precipitated overnight at -70°C. The next day, the tubes were centrifuged at 12000 rpm for 10 min and the pellet was washed with 1 volume of ethanol 70°. After centrifugation at 8000 rpm for 5 min and discarding the supernatant, the pellet was dried at RT and re-eluted in 40 ul of nuclease-free water. RNAse was added to enhance the purification of genomic DNA.
